# Geminate labels programmed by two-tone microdroplets combining structural and fluorescent color

**DOI:** 10.1038/s41467-021-20908-y

**Published:** 2021-01-29

**Authors:** Lang Qin, Xiaojun Liu, Kunyun He, Guodong Yu, Hang Yuan, Ming Xu, Fuyou Li, Yanlei Yu

**Affiliations:** 1 Department of Materials Science and State Key Laboratory of Molecular Engineering of Polymers, Fudan University, Shanghai, 200433 P. R. China; 2 Department of Chemistry and State Key Laboratory of Molecular Engineering of Polymers, Fudan University, Shanghai, 200433 P. R. China

**Keywords:** Optical materials, Photonic crystals, Liquid crystals

## Abstract

Creating a security label that carries entirely distinct information in reflective and fluorescent states would enhance anti-counterfeiting levels to deter counterfeits ranging from currencies to pharmaceuticals, but has proven extremely challenging. Efforts to tune the reflection color of luminescent materials by modifying inherent chemical structures remain outweighed by substantial trade-offs in fluorescence properties, and vice versa, which destroys the information integrity of labels in either reflection or fluorescent color. Here, a strategy is reported to design geminate labels by programming fluorescent cholesteric liquid crystal microdroplets (two-tone inks), where the luminescent material is ‘coated’ with the structural color from helical superstructures. These structurally defined microdroplets fabricated by a capillary microfluidic technique contribute to different but intact messages of both reflective and fluorescent patterns in the geminate labels. Such two-tone inks have enormous potential to provide a platform for encryption and protection of valuable authentic information in anti-counterfeiting technology.

## Abstract (web summary)

Creating an enhanced-security-level label that carries entirely distinct information in different optical states has proven challenging. Here, the authors design geminate labels by programming fluorescent cholesteric liquid crystal microdroplets to encrypt fluorescent security information behind colorful reflective patterns.

## Introduction

Anti-counterfeiting technology, in which security labels are popular elements for protecting authentic articles, is of significance to help companies, customers and governments reduce economic loss threatened by counterfeit goods in paper currency, medicine, edible, clothing, and microelectronics^1,2^. The cutting edge in the development of next-generation anti-counterfeiting technologies currently focuses on designing security inks that are able to offer numerous optical states to convey distinct information. To this end, researchers strive to develop novel inks of luminescent materials as carriers of authentic messages, including lanthanide-doped nanoparticles, semiconducting quantum dots, organic dyes, and metallic nanoparticles, by tuning fluorescent color, intensity, and lifetime value^3–9^. As another color of the inks, reflection color arising from the selective absorption of visible light by pigments (pigment color), however, has rarely been taken into consideration in security labels with fluorescent color. If both reflection and fluorescent colors are simultaneously programmed to design a geminate label, which is capable of demonstrating entirely different information in reflective and fluorescent states, the reflection color will also be adopted to offer more optical states for carrying information, thus enhancing the anti-counterfeiting level of the security labels. Although researchers have reported plenty of paradigms to tune the fluorescence for encoding intricate information, it is a great challenge to arbitrarily program the reflective patterns while maintain the integrity of the fluorescent patterns, and vice versa. The limitation lies in that efforts to tune the reflection color of luminescent materials by modifying inherent chemical structures would inevitably change the fluorescence properties at the same time, because both of them are determined by the molecular structures (organic) or energy level structures (inorganic) of the pigments.

Nature, however, provides an alternative strategy to generate reflection color that has nothing to do with the pigments but capitalizes on photonic crystals: instead of using chemical or energy level structures to selectively absorb light, systems, such as *Morpho* butterflies, *Cotinga maynana* birds and *Pollia condensata* fruits, use periodic structures to selectively reflect light, commonly referred to as structural color^10–13^. Inspired by this phenomenon, we hypothesize that the structural color could be used as a “coating” to replace the pigment color of luminescent materials. In this scenario, the structural color acts as reflection color that could be tuned by altering the periodic structures of the photonic crystals whereas the fluorescent color, on their own, remains the same. To test this hypothesis, an ideal candidate of photonic crystals to realize this goal is cholesteric liquid crystal(s) (CLCs), where the molecules are parallelly aligned in each layer and slightly twisted along the normal direction of layers in a helical fashion^14–20^. One of the intriguing features of CLCs is to selectively reflect the circularly polarized light, according to Bragg’s law, with the same handedness as the helix. This unique soft photonic crystal material can be easily prepared and constructed into multidimensional architectures from planar to spherical periodic structures^21–33^.

**Fig. 1:**
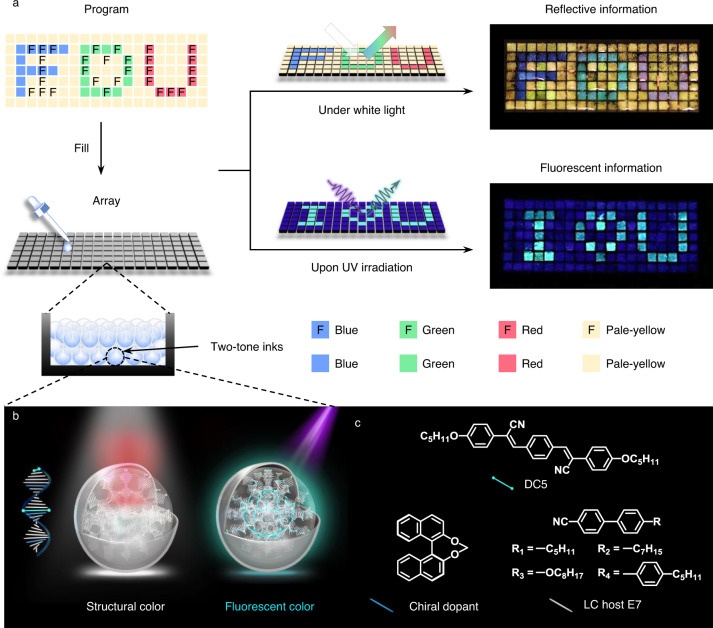
Design of geminate labels programmed by two-tone microdroplets. **a** Schematic illustration to show the design of a geminate label created by using two-tone inks and photographs of the resulting label (47 mm × 17 mm) in reflective and fluorescent states. The label carrying two distinct kinds of information demonstrates a reflective pattern of RGB “FDU” under white light and a fluorescent pattern of cyan “I love U” upon UV irradiation. The blue, green, red, and pale-yellow colors of the blocks represent the reflection colors of the microdroplets and letter “F” represents the microdroplets with fluorescence. **b** Schematic illustration to show fluorescent cholesteric liquid crystal (FCLC) microdroplets (two-tone inks) combining structural color under white light and fluorescent color upon UV irradiation. **c** Chemical structures of the materials used to prepare the FCLC mixtures, including fluorescent molecule DC5, (S)-binaphthyl derivative chiral dopant and LC host E7.

Herein, a geminate label carrying two distinct kinds of information, for example, RGB letters “FDU” in reflective state and a cyan “I love U” pattern in fluorescent state, is programmed and created in an array by fluorescent CLC (FCLC) microdroplets, which we name “two-tone” inks (Fig. 1a). It should be emphasized that the “two-tone” refers to two colors of different mechanisms: the fluorescent color is still determined by the molecular structure of the pigment while the structural color is controlled by the pitch length of helical superstructures in CLCs (Fig. 1b). Hence, the FCLC microdroplets can exhibit rich reflection colors under white light and fluorescent color upon UV irradiation. We demonstrate the potential of our FCLC microdroplets as two-tone inks to program enhanced-security-level geminate labels in anti-counterfeiting technology.

## Results

The FCLC microdroplets (two-tone inks) were designed based on two criteria: (1) the fluorescence must be introduced into the CLC system, and (2) each ink must be uniform in size where the helical superstructures are well aligned. The first requirement is satisfied by doping a fluorescent molecule DC5 and chiral dopant into the nematic LC host E7 (Fig. 1c; see Supplementary Fig. 1 and 2 for synthetic routes). Considering the self-organization nature of LCs, we synthesized a cyano-substituted oligo(*p*-phenylene vinylene) derivative DC5 as the fluorescent molecule due to its intense emission in both solution and solid state to avoid aggregation-caused quenching (ACQ) effect^34–38^. The peripheral flexible *n*-pentyloxy groups (-OC_5_H_11_) together with central rigid core are designed to mimic the structure of LC molecules, increasing the solubility of DC5 in LC host E7 to prepare homogeneous fluorescent LC mixtures. The bridged (S)-binaphthyl chiral dopant with high helical twisting power (HTP) is chosen to twist the nematic LC into helical superstructures. To satisfy the second criterion, flow-focusing glass capillary microfluidic is a promising technique, by which monodisperse microdroplets with radially aligned helices are continuously fabricated in a controlled fashion with the aid of certain surfactants^39–42^. From these principles, we fabricated a set of FCLC microdroplets aiming at the creation of geminate labels with distinct patterns in reflection and fluorescent color.

**Fig. 2:**
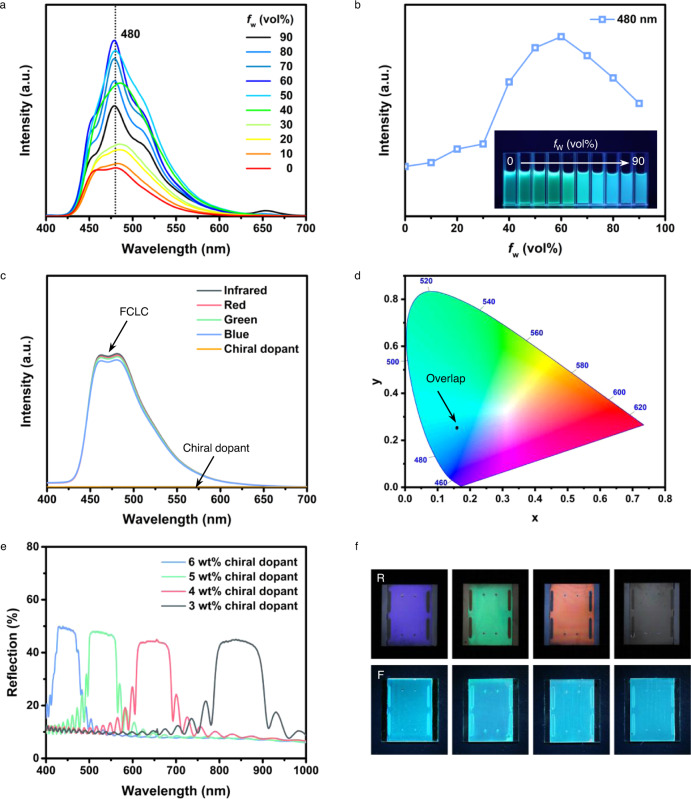
Characterization of the FCLCs. **a** Photoluminescence (PL) spectra of DC5 in THF and THF/water mixtures (*c* = 10^−4^ M). The excitation wavelength is 388 nm. **b** A Plot of the emission intensity at 480 nm versus water fractions (*f*_w_) for DC5 in THF and THF/water mixtures. The inset photograph shows DC5 in THF and THF/water mixtures (*f*_w_ increases from 0 to 90 vol%) upon UV irradiation. **c** PL spectra of FCLC mixtures containing 6, 5, 4, 3 wt% chiral dopants in THF (*c* = 0.5 M) and chiral dopant in THF (*c* = 10^−4^ M). **d** CIE chromaticity coordinates of different FCLC mixtures containing 6, 5, 4, 3 wt% chiral dopants in THF (*c* = 0.5 M), which are (0.159, 0.251), (0.159, 0.252), (0.159, 0.254), and (0.159, 0.254), respectively. **e** Reflectance spectra of the FCLC mixtures containing 6, 5, 4, 3 wt% chiral dopants in 5 μm thick antiparallel aligned cells. **f** Photographs of the FCLC mixtures containing 6, 5, 4, 3 wt% chiral dopants in 5 μm thick antiparallel aligned cells to show blue, green, red, transparent (infrared) reflection colors and cyan fluorescent color. R, reflection color under white light; F, fluorescent color upon UV irradiation.

It is important to note that the LC is a kind of fluid possessing a certain viscosity, where molecular self-organization or aggregation is an intrinsic natural process. Therefore, traditional luminescent materials either have poor solubility or suffer from the ACQ effect when aggregates are formed^43^. DC5 is a promising candidate to solve these problems, because it is completely free of ACQ effect and emits intense light with high fluorescence quantum yields in both solution (85.7% in THF) and solid states (up to 100%) (Supplementary Fig. 3). On the basis of the UV-vis spectrum (Supplementary Fig. 4), photoluminescence (PL) spectra of DC5 in THF and THF/water mixtures were excited at 388 nm (absorption maximum) and comprehensively investigated to testify the visual observation (Fig. 2a, b). As shown in Fig. 2a, DC5 emits cyan light with emission maximum at 480 nm when the fraction of poor solvent water (*f*_w_) increases to cause aggregation of DC5 in the aqueous mixtures. The increase of PL intensity at 480 nm (*f*_w_ ≤ 60%) is the consequence of aggregation-induced emission (AIE) effect of DC5 that aggregates into suspension as crystal flakes shown in Supplementary Fig. 5. When the *f*_w_ is higher than 60%, the PL intensity of DC5 with the same lattice structure (Supplementary Fig. 6) gradually decreases. This phenomenon may be ascribed to the reduced number of emitting molecules because only the molecules on the surface of the suspensions emit light upon excitation after the aggregation, leading to a decrease in PL intensity^44,45^. Although the PL intensity varies from the molecular solution in THF to nanosuspension in 10/90 THF/water mixture (Fig. 2b), DC5 emits intense fluorescence in all states upon UV irradiation, which is directly evidenced by the inset in Fig. 2b, indicating that DC5 is qualified as the luminescent material in the LC system.

The homogeneous fluorescent LC mixture was prepared by doping a very small amount of DC5 (0.25 wt‰) into commercially available nematic LC host E7 because of the high fluorescence quantum yield. The DSC curves obviously indicate that our eutectic fluorescent LC mixture retains the basic features of the LC host E7, including the clearing temperature (*T*_c_ = 60 °C), a wide temperature range of LC phase, and notably the nematic phase at room temperature that can be readily induced to form the CLC phase (Supplementary Fig. 7). We further demonstrated that, by using wedge cells according to the Grandjean-Cano method^46^, the chiral dopant shows an HTP value as high as 66 μm^−1^ (wt%) in the fluorescent LC mixture (Supplementary Fig. 8). A series of mixtures containing 6, 5, 4, 3 wt% chiral dopants in the fluorescent LC were proved to emit uniform cyan fluorescence with similar intensities (Fig. 2c), whose corresponding CIE chromaticity coordinates were almost overlapped with each other in Fig. 2d. Moreover, these mixtures were filled into 5 μm thick antiparallel aligned cells and reflected wavelengths of 445, 530, 650, and 880 nm, respectively (Fig. 2e). Accordingly, the mixtures confined in cells to form one-dimensional photonic crystal structures exhibit blue (445 nm), green (530 nm), red (650 nm), and transparent (infrared reflection at 880 nm is invisible) reflection colors under white light, and, importantly, cyan fluorescent color upon UV irradiation (365 nm, 10 mW/cm^2^; Fig. 2f). In addition to tunning the structural color by adding different weights of chiral dopants into the fluorescent LC host, various structural colors were also obtained by mixing the blue and infrared FCLC mixtures in different proportions, whose fluorescent color is uniform cyan (Supplementary Fig. 9). These experimental results clearly demonstrate that the FCLC mixtures are successfully prepared according to our facile and flexible method, in which the goal is realized by “coating” the fluorescent color with different structural colors as designed.

**Fig. 3:**
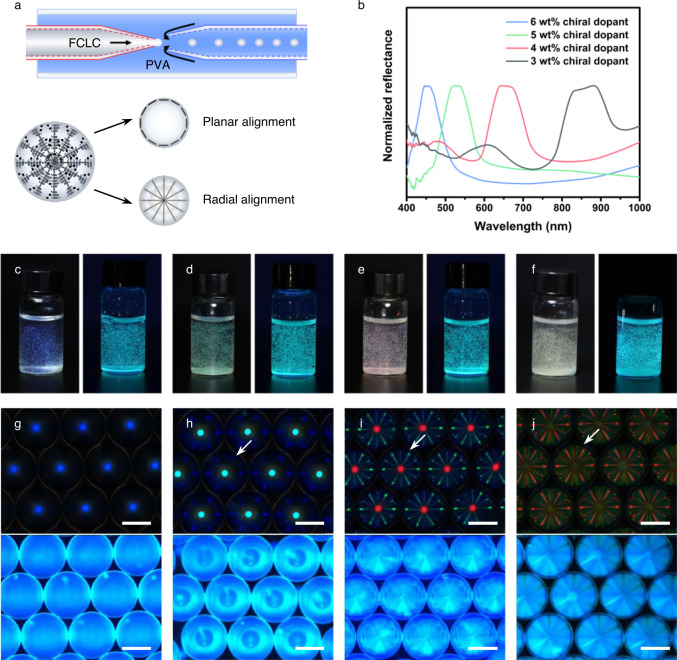
FCLC microdroplets fabricated by capillary microfluidic technique. **a** Schematic illustration to show the capillary microfluidic device used to fabricate the FCLC microdroplets. The hydrophobic and hydrophilic surfaces of the capillaries are denoted in red and blue. The 5 wt% PVA aqueous solution is used as continuous phase to facilitate planar alignment of the LC molecules and radial alignment of the helical axes. **b** Normalized reflectance spectra of the FCLC microdroplets containing 6, 5, 4, 3 wt% chiral dopants, respectively. **c**–**f** Photographs of blue, green, red, and infrared FCLC microdroplets to show striking reflection colors under white light and fluorescent color upon UV irradiation. **g**–**j** POM images of blue, green, red, and infrared monodisperse FCLC microdroplets in hexagonally close-packed arrays to show photonic cross-communication and fluorescent patterns. Additional lines caused by double reflection are denoted by white arrows. The scale bar is 100 μm.

The FCLC mixtures with random aligned helices are inadequate for two-tone inks owing to their dim structural colors; therefore, they are further processed into structurally defined and monodisperse microdroplets by using flow-focusing glass capillary microfluidic technique with the aid of an appropriate surfactant^47^. The device is composed of two kinds of glass capillaries that are coaxially assembled: the left capillary is hydrophobic (denoted in red) to ensure the surface is wetted by the FCLC mixtures, while the right capillary features hydrophilicity (denoted in blue) to prevent the sticking of microdroplets on the surface (Fig. 3a; the details are described in Methods). Both the FCLC mixtures and 5 wt% aqueous solution of poly(vinyl alcohol) (PVA) are simultaneously injected through the orifice of the collection capillary to fabricate monodisperse microdroplets (Supplementary Movie 1). In these microdroplets, the helical axes are radially aligned and perpendicular to the surface, which is attributed to the PVA as a surfactant in continuous phase that facilitates planar alignment of the LC molecules^48^. As the reflectance spectra shown in Fig. 3b, the central reflection wavelengths of blue, green, red, and infrared microdroplets are consistent with those of the FCLC mixtures confined in the antiparallel aligned cells, indicating that the pitch lengths remain constant during the fabrication (Supplementary Fig. 10). As a result, the well-aligned FCLC microdroplets exhibit striking reflection colors under white light and cyan fluorescent color upon UV irradiation as well (Fig. 3c–f). We note that the infrared microdroplets, however, exhibit pale-yellow reflection color originating from the pigment color of DC5 rather than the structural color of CLCs, because invisible infrared reflection at 880 nm serves as a “transparent coating”, and hence the pigment color of DC5 becomes visible in this scenario (Fig. 3f).

## Discussion

To gain further insight into the structural and fluorescent colors, a single-layer array of hexagonally close-packed monodisperse FCLC microdroplets was investigated by using polarized optical microscope (POM) (Fig. 3g–j). In reflection mode, unique patterns of structural color were observed due to Bragg’s law, *λ* = *np*cos*θ*, where *θ* is the angle between the direction of light propagation and helical axes. The central dots in eaWangch microdroplet are caused by normal reflection (*θ* = 0°), whereas the additional radial lines between neighboring microdroplets are caused by double reflection (*θ* = 45°) (Supplementary Fig. 11 and 12). The reflected light from one microdroplet exactly acts as the incident light for neighboring microdroplets, and subsequently is reflected again according to Bragg’s law, which is known as photonic cross-communication^27,49^. In fluorescence mode, special patterns of the fluorescent color were observed in the hexagonally close-packed arrays upon UV irradiation. Interestingly, the ordered FCLC microdroplets demonstrate unexpected patterns, including pearls, yolk shells, hexagonal nests, and wheels, which are different from those of the photonic cross-communication and relate to the pitch length of the helical superstructures as well as inherent emission in microdroplets. These inimitable patterns indirectly indicate the radial configuration of the helical axes and planar alignment of the LC molecules along the spherical surface^50^.

The diameters of the FCLC microdroplets were controlled by adjusting the volumetric flow rates of the continuous phase (Q_c_) to operate at dripping mode. When the volumetric flow rate of the dispersed phase (*Q*_d_) is fixed at 60 μL h^−1^, the diameters of the microdroplets at any concentration of the chiral dopant decrease from ~214 to ~144 μm as *Q*_c_ increases from 1000 to 5000 μL h^−1^ (Table 1). The microdroplets possess strictly uniform sizes with coefficients of variation all less than 0.87% (Supplementary Fig. 13). Furthermore, the microdroplets with the same pitch length but different sizes exhibit the same structural and fluorescent color (Supplementary Fig. 14), and remain the original features (size, structural and fluorescent color) in PVA aqueous solution for at least 40 days (Supplementary Fig. 15). Under continuous 365 nm UV irradiation for 1 h, the PL intensity of the FCLC microdroplets slowly decreased to 83.6%, indicating that the two-tone microdroplets have acceptable fatigue resistance (Supplementary Fig. 16).

**Table 1:** Flow rate dependence of the average diameter (d_ave_) of the monodispersed FCLC microdroplets.

*C*_chiral_ (wt%)	3		4		5		6	
*Q*_c_ (μL h^−1^)	*d*_ave_ (μm)	CVs (%)	*d*_ave_ (μm)	CVs (%)	*d*_ave_ (μm)	CVs (%)	*d*_ave_ (μm)	CVs (%)
1000	210	0.39	213	0.25	216	0.32	215	0.24
2000	185	0.53	184	0.23	187	0.41	188	0.43
3000	173	0.50	171	0.43	170	0.44	168	0.68
4000	154	0.81	156	0.56	159	0.53	157	0.41
5000	146	0.51	147	0.58	142	0.56	141	0.87

Average diameters and coefficients of variation (CVs) of the FCLC microdroplets at different volumetric flow rates of the continuous phase (*Q*_c_). The volumetric flow rate of the dispersed phase (*Q*_d_) is set to be 60 μL h^−1^.

**Fig. 4:**
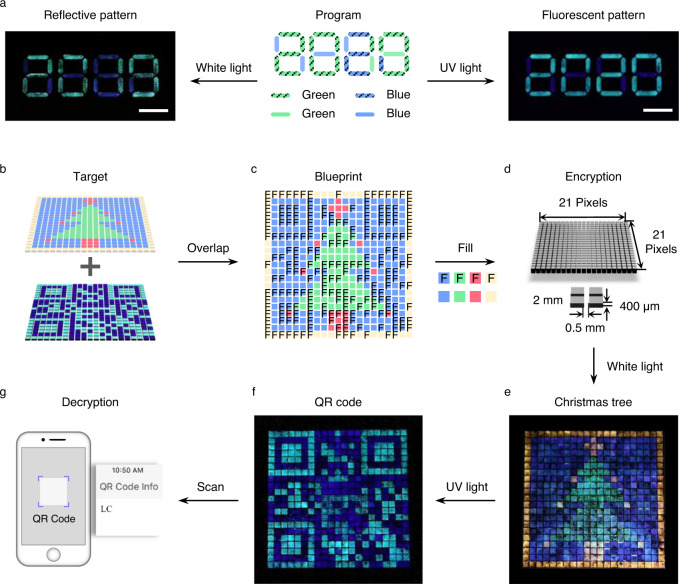
Geminate labels carrying two distinct kinds of information. **a** A geminate label with two different messages of a green “2019” reflective pattern and a cyan “2020” fluorescent pattern. In program, the color of the rectangles represents the reflection colors of the microdroplets and the slashes in the rectangles represent the microdroplets with fluorescence. The scale bar is 1 cm. **b**–**g** Encryption and decryption of an enhanced-security-level geminate label created by eight kinds of microdroplets in a “pixelated” array (21 × 21). The fluorescent QR code of “LC” is concealed behind a colorful reflective “Christmas tree”, which can be decrypted by smartphones upon UV irradiation. Each pixel is 2.0 mm × 2.0 mm × 400 μm, and the distance between two pixels is 0.5 mm.

Both the FCLC microdroplets and CLC microdroplets without fluorescence (fabricated by the same capillary microfluidic technique; Supplementary Fig. 17) are programmed to create geminate labels that carry two distinct kinds of information. Since the diameter of the microdroplets has a negligible effect on visual observation (Supplementary Fig. 18), we used the microdroplets with a diameter of ~170 μm in the following experiments (*Q*_c_ = 3000 μL h^−1^, *Q*_d_ = 60 μL h^−1^). First, a simple label was created as depicted in the program by using the FCLC and CLC microdroplets with blue reflection color (Supplementary Fig. 19). The label demonstrates a blue reflective pattern of the square and a cyan fluorescent pattern of inverted Chinese character “Fu” as expected, because both the FCLC and CLC microdroplets exhibit blue reflection color under white light, while only the FCLC microdroplets exhibit cyan fluorescent color upon UV irradiation. Furthermore, an intricate geminate label with two different messages of reflective green “2019” and fluorescent cyan “2020” was created by using the FCLC and CLC microdroplets with blue and green reflection colors (Fig. 4a). The structural color of the labels under white light is angular-independent, owing to the omnidirectional Bragg reflections produced by the three-dimensional microdroplets (Supplementary Fig. 20)^25^. Guo and Li et al. recently have reported an interesting dual-mode CLC system induced by light-driven fluorescent chiral switches, in which the reflection wavelength and fluorescence intensity were simultaneously tuned due to the photoisomerization of the chiral switch, and demonstrated only one pattern in both the reflective and fluorescent modes^51^. However, our designed two-tone inks allow creating geminate labels with totally different reflective and fluorescent patterns.

To test our hypothesis of enhanced anti-counterfeiting level, we further create a geminate “security label” by using two-tone inks and demonstrate the proof of concept as schematically depicted in Fig. 4b, c. The label is designed according to the following process. (1) Choose reflective and fluorescent patterns, for example, we want to demonstrate a reflective “Christmas tree“ and a fluorescent QR code of “LC” (Fig. 4b), (2) overlap each other to generate a blueprint that illustrates the required inks (Fig. 4c), and (3) fill an array according to the blueprint. Here, a “pixelated” black array (21 × 21) of polymethyl methacrylate (PMMA) was fabricated by computerized numerical control engraving machine (each pixel is 2 mm × 2 mm × 400 μm) and filled with eight kinds of microdroplets serving as security inks based on the blueprint (Fig. 4d). Consequently, the geminate label demonstrates the desired colorful “Christmas tree” in the reflective state under white light (Fig. 4e), and changes into cyan QR code of “LC” in fluorescent state upon UV irradiation, which can be decrypted by smartphones in an optimal distance of 20–30 cm (Fig. 4f, g; Supplementary Movie 2 and 3). With this strategy, the geminate labels are hidden in colorful packaging of the goods by programming the structural color of the microdroplets, which are difficult to be recognized under white light. Whereas the specific patterns encrypted in the fluorescent state of the labels will appear upon UV irradiation. The size, pattern, and position of the authentic information concealed behind colorful reflective coatings or decorations could be designed without destroying their integrity, which demonstrates that our two-tone inks have promise for application in anti-counterfeiting technology.

In summary, we proposed a conceptually novel strategy to construct two-tone inks by replacing the pigment color of luminescent materials with the structural color from photonic crystal “coating”. The fluorescence was successfully introduced into the CLC mixtures by doping a very small amount of newly designed DC5 (0.25 wt‰) with high fluorescence quantum yields in both solution and solid states. The FCLC mixtures were further processed into structurally defined and monodisperse microdroplets by capillary microfluidic technique, which exhibited striking reflection colors and bright fluorescent color. Therefore, geminate labels were programmed by using such FCLC microdroplets as two-tone inks to demonstrate two kinds of entirely distinct but intact patterns in reflective and fluorescent states. According to the design, one can encrypt any security information, such as QR codes, in fluorescent patterns that are concealed behind colorful reflective patterns to deter counterfeits. This two-tone ink opens new opportunities for the combination of two fundamental optical features (reflection and fluorescence) and has enormous potential to greatly enhance the anti-counterfeiting level of security labels, providing a versatile platform for encryption and protection of valuable authentic information in anti-counterfeiting technology.

## Methods

### Materials

All chemical reagents were purchased from Adamas-beta and were used as supplied without further purification. The nematic LC host E7 (*n* = 1.747, *T*_c_ = 60 °C) was purchased from Nanjing Murun Advanced Material Co., Ltd. The preparation of the chiral dopant and the fluorescent molecule DC5, and the HTP measurement of the chiral dopant are described in the Supplementary Information.

### Preparation of the FCLC and CLC mixtures

0.25 wt‰ fluorescent molecule DC5 and LC host E7 were mixed in dichloromethane solution to prepare the fluorescent LC mixture after evaporation of the solvent. 3, 4, 5, and 6 wt% chiral dopants were added in the fluorescent LC host to prepare the infrared, red, green, and blue FCLC mixtures, respectively. Similarly, 3, 4, 5, and 6 wt% chiral dopants were added in LC host E7 to prepare the infrared, red, green, and blue CLC mixtures, respectively.

### Fabrication of the FCLC and CLC microdroplets

The microfluidic device was composed of two types of glass capillaries, where two smaller capillaries were coaxially assembled into a bigger one. The inner capillaries were tapered with a Sutter Instrument P-1000 micropipette puller and polished to the desired diameters. Here, the left capillary with an orifice diameter of 110 μm was treated with octadecyltriethoxysilane (TCI Development Co., Ltd.) to become hydrophobic, and the right capillary with an orifice diameter of 240 μm was treated with Piranha solution (H_2_SO_4_:H_2_O_2_ = 7/3, v/v) to become hydrophilic. The FCLC or CLC mixtures as the inner phase were dispersed into 5 wt% aqueous solution of PVA (M_w_ = 13,000–23,000 g mol^−1^) as the outer phase to fabricate the monodisperse microdroplets. The diameter of the microdroplets was controlled by the flow rates of the outer phases using Lead Fluid TYD01 syringe pumps, and the flow rate of inner phase was fixed at 60 μL h^−1^. Fabrication of the microdroplets was monitored by an optical microscopy.

### Fabrication of the geminate labels

The 21 × 21 “pixelated” black array of PMMA was fabricated by computerized numerical control engraving machine. The size of each pixel was 2 mm × 2 mm × 400 μm. After the collection of the FCLC or CLC microdroplets fabricated by the glass capillary microfluidic device, the excess PVA aqueous solution was removed to concentrate the density of microdroplets in the vial. Then the FCLC and CLC microdroplets were selectively drawn to fill the array by using glass droppers that were treated with Piranha solution (H_2_SO_4_:H_2_O_2_ = 7/3, v/v) to become hydrophilic. Particularly, the orifice diameter of the glass dropper should be smaller than the size of the pixels (2 mm).

### Measurements

^1^H NMR and ^13^C NMR spectra were recorded on a Bruker AVANCE III spectrometer at 400 MHz with use of CDCl_3_ as the lock. Melting points were measured on a TA Q2000 differential scanning calorimeter. Mass spectra were recorded on an AB SCIEX 5800 mass spectrometer. Fluorescence spectra and photobleaching property were recorded on an Edinburgh Instruments FS5 with an integrating sphere. The surface morphology of the DC5 in THF and THF/water was examined by a FESEM Zeiss Ultra 55. 2D WAXD experiments were conducted on a Bruker D8 Discover diffractometer with a 2D detector of general area detector diffraction system (GADDS) in the transmission mode. Fluorescence lifetime was measured on an Edinburgh Instruments FLS1000. Reflection spectra were recorded on an Ideaoptics Instruments PG2000-Pro-EX reflection spectrometer (200–1100 nm). The cross-communication in a single-layer array of hexagonally close-packed monodisperse microdroplets and the disclination lines in the wedge cell were observed by a Leica DM2500P POM. Wedge cells (KCRK-07) and antiparallel aligned cells were purchased from EHC Co., Ltd. 365 nm UV light was generated by an Omron ZUV-H30MC light source with a ZUV-C30H controller (10 mW/cm^2^).

## Supplementary information

Supplementary Information

Description of Additional Supplementary Files

Supplementary Movie 1

Supplementary Movie 2

Supplementary Movie 3

## Data Availability

The authors declare that the main data supporting the findings of this study are available within the article and its Supplementary Information files. Extra data are available from the corresponding author upon reasonable request.
