# Supplementary material for: Geminate labels programmed by two-tone microdroplets combining structural and fluorescent color
Source: Nat Commun. 2021 Jan 29;12:699. doi: 10.1038/s41467-021-20908-y (PMC7846849; doi:10.1038/s41467-021-20908-y)
Supplement: Supplementary file 1 — Supplementary Information [file 41467_2021_20908_MOESM1_ESM.pdf]

Supplementary Information

Geminate labels programmed by two-tone microdroplets combining structural and fluorescent color

Lang Qin[1,#], Xiaojun Liu[1,#], Kunyun He[1], Guodong Yu[1], Hang Yuan[2], Ming Xu[2], Fuyou Li[2], and Yanlei Yu[1,*]

[1]Department of Materials Science & State Key Laboratory of Molecular Engineering of Polymers, Fudan University, Shanghai 200433, China

[2]Department of Chemistry & State Key Laboratory of Molecular Engineering of Polymers, Fudan University, Shanghai 200433, China

[#]These authors contributed equally to this work.

*Email: ylyu@fudan.edu.cn

1. Synthesis

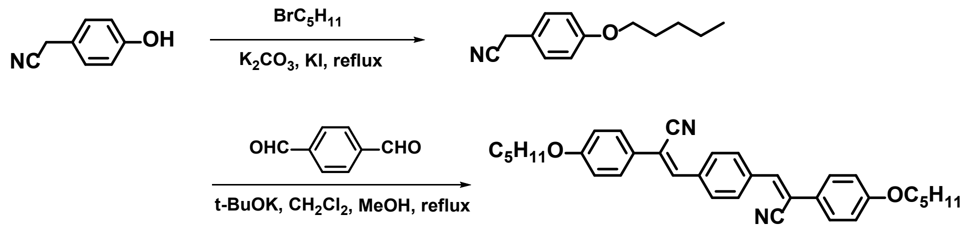

Supplementary Fig. 1 Synthetic route of fluorescent molecule DC5.

4-cyanomethylphenol (3.0 g, 22.53 mmol), 1-bromobutane (4.0 g, 26.5 mmol), K_2CO_3 (3.7 g, 26.5 mmol) and potassium iodide were added in 2-butanone (20 mL). The mixture was heated to 80 °C for 6 h, and then washed with water. After extracted with dichloromethane, the combined organic layers were dried over $MgSO_4$ and concentrated. The crude product was purified by column chromatography (PE/CH_2Cl_2, 80/20) on silica gel, which is colorless transparent liquid (3.6 g, 78.8%). The intermediate was used in next step at once. The intermediate (1.51 g, 7.46 mmol) and terephthalaldehyde (0.5 g, 3.73 mmol) was dissolved in dichloromethane (10 mL). The mixture was heated to 50 °C. A solution of potassium tert-butoxide (0.83 g, 7.46 mmol) in methanol (10 mL) was added dropwise with stirring. After the reaction was completed, the crude product was purified by column chromatography with dichloromethane on silica gel. A fluorescent yellow solid was obtained by recrystallization in methanol (1.2 g, 63.7%). M.p. 184 °C. ^{1}H NMR (400 MHz, $CDCl_3$): δ 7.95 (s, 4H), 7.62 (d, J = 8.8 Hz, 4H), 7.42 (s, 2H), 6.96 (d, J = 8.8 Hz, 4H), 4.01 (t, J = 13.2 Hz, 4H), 1.84-1.78 (m, 4H), 1.50-1.36 (m, 8H), 0.95 (t, J = 14.4 Hz, 6H). ^{13}C NMR (400 MHz, $CDCl_3$): δ 160.34, 138.38, 135.40, 129.50, 127.43, 126.50, 118.00, 115.04, 112.35, 68.27, 28.87, 28.17, 22.45, 14.02; HRMS (MALDI-TOF): calcd. for $C_{34}H_{36}N_2O_2^+$ $[M + H]^+$ 505.28, found 505.26.

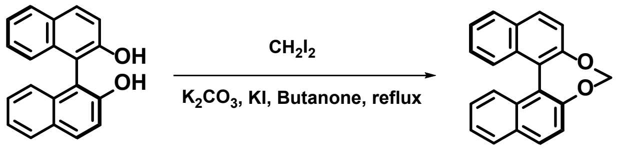

Supplementary Fig. 2 Synthetic route of chiral dopant.

A mixture of (S)-(1,1'-binaphthalene)-2,2'-diol (3.0 g, 8.8 mmol), diiodomethane (2.83 g, 10.6 mmol), K_2CO_3 (1.46 g, 10.6 mmol) and potassium iodide in 2-Butanone (30 mL) was stirred magnetically and refluxed at 85 °C for 5 h. After cooling to room temperature, the reaction mixture was washed with water, extracted by dichloromethane, and then purified by column chromatography (PE/CH_2Cl_2, 80/20) on silica gel. The product was recrystallized in methanol to yield white solid (1.65 g, 62.7%). M.p. 193 °C. ^{1}H NMR (400 MHz, $CDCl_3$): δ 7.92 (d, J = 8.4 Hz, 4H), 7.49 (d, J = 8.8 Hz, 2H), 7.44 (t, J = 8 Hz, 2H), 7.42 (t, J = 8 Hz, 2H), 7.31-7.26 (m, 2H), 5.68 (s, 2H). ^{13}C NMR (400 MHz, $CDCl_3$): δ151.35, 132.25, 131.87, 130.40, 128.47, 126.98, 126.41, 125.07, 121.03, 103.21; HRMS (MALDI-TOF): calcd. for $C_{21}H_{14}O_2^+$ $[M + H]^+$ 299.10, found 298.98.

2. Characterization of fluorescent molecule DC5

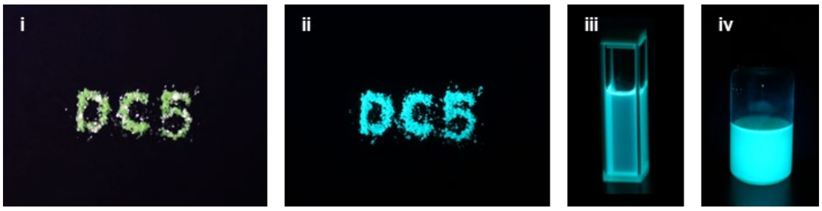

Supplementary Fig. 3 Photographs of DC5 crystal under white light (i), upon UV irradiation (ii), and in THF solution (c = 10^{-4} M) upon UV irradiation (iii). A Photograph of fluorescent LC mixture (0.25 wt ‰ DC5 in LC host E7) upon UV irradiation (iv).

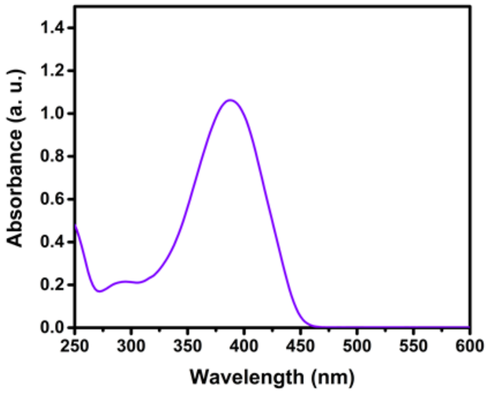

Supplementary Fig. 4 An UV-vis spectrum of DC5 in dichloromethane (10^{-5} M). The absorption maximum is 388 nm.

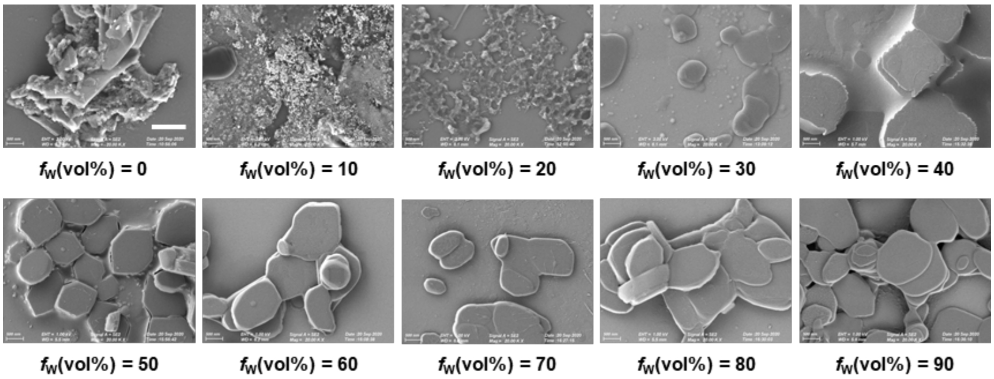

Supplementary Fig. 5 SEM images of DC5 in THF and THF/water mixtures (c = 10^{-4} M), where the f_w increases from 0 to 90 vol%. The scale bar is 1 μm.

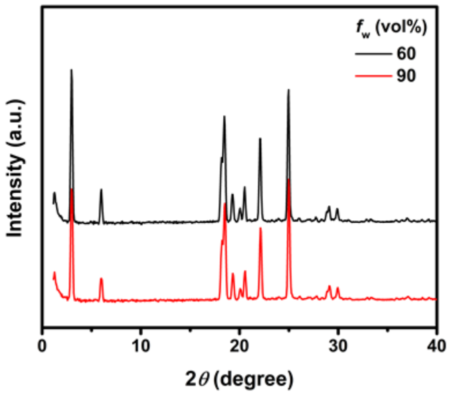

Supplementary Fig. 6 WXRD patterns of DC5 suspensions in 40/60 and 10/90 THF/water mixtures.

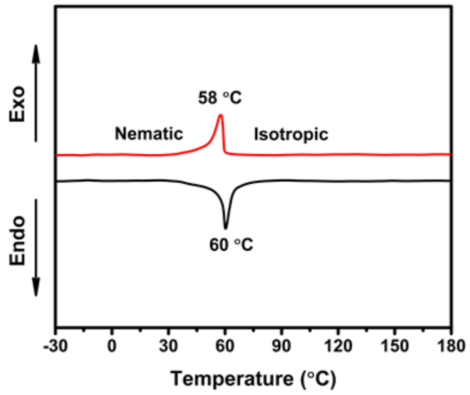

Supplementary Fig. 7 DSC curves of the fluorescent LC mixture (0.25 wt ‰ DC5 in LC host E7).

3. Measurement of helical twisting power (HTP)

The HTP value describes the ability of the chiral dopant to twist nematic LC, which is determined by $\beta = (pc)^{-1}$, where β is the helical twisting power, p is the pitch of CLC, and c is the concentration of the chiral dopant.

According to the conventional technology, Grandjean-Cano wedge method [1], pitch can be measured. The used wedge cell with an opening angle is made by applying two different-sized spacers at each end of the cell. If the alignment of the substrates is planar (the director lies parallel to the surface) and the rubbing directions of substrates are parallel to one another, the CLC becomes discrete. Because the value of the pitch is fixed, and the alignment is also fixed, the CLC arranges itself as shown in Figure S8. This arrangement results in disclination lines between areas that contain a different number of layers. The difference in thickness between each domain must be $p/2$ in order to satisfy the alignment boundary condition. By using a POM, the disclination lines of the CLC in the wedge cell can be seen. The pitch was determined according to the equation $p = 2R\tan\theta$, where R represents the distance between the disclination lines and θ is the wedge angle of wedge cells (EHC, KCRK-07, $\tan\theta = 0.0183$).

The concentration of the chiral dopant is 0.70 wt%. According to the equation mentioned above, the HTP of the chiral dopant in the fluorescent LC mixture is 66 μm^{-1} (wt%).

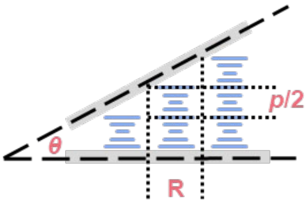

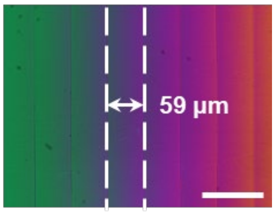

Supplementary Fig. 8 Schematic illustration showing a wedge cell for pitch measurement of the CLC mixture (left) and measurement of disclination lines of 0.70 wt% chiral dopant in the fluorescent LC mixture (right). The disclination lines are pointed out with white lines. The scale bar is 100 μm.

4. Structural and fluorescent color of the FCLC mixtures

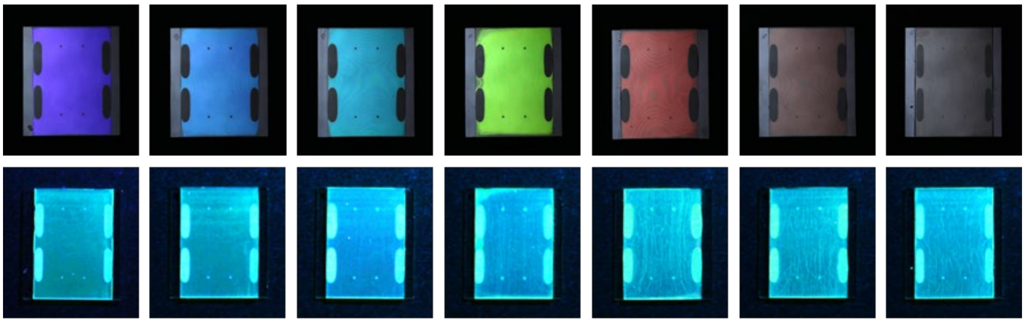

Supplementary Fig. 9 Photographs of the FCLC mixtures in 5 μm thick antiparallel aligned cells to show various structural colors under white light (top) and uniform cyan fluorescent color upon UV irradiation (bottom). The FCLC mixtures were obtained by mixing the blue and infrared FCLC mixtures in different proportions. The reflection wavelengths are 440, 475, 490, 565, 670, 712, and 810 nm from left to right. [a]Mass ratio of the blue and infrared FCLC mixtures.

5. The FCLC microdroplets and photonic cross-communication

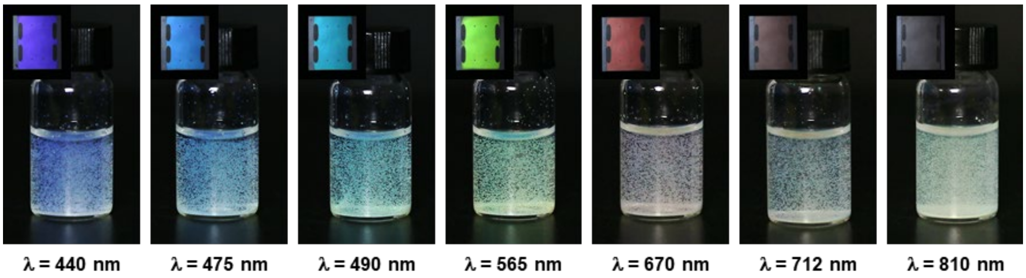

Supplementary Fig. 10 Photographs of the FCLC microdroplets to show structural colors under white light. The structural colors of the microdroplets are the same as that of the corresponding mixtures in 5 μm thick antiparallel aligned cells (the inset photographs).

The special patterns of reflection colors in the FCLC microdroplets arrays are attributed to the angle θ between the incident light and the helical axes, as shown in Figure S11. When the θ is 0°, the incident light is normally reflected as depicted by the black arrow. When the θ is 45°, the incident light was reflected by one microdroplet to propagate to the neighbors, and then reflected again to be parallel to the incident light, as demonstrated by red arrows. This double reflection between the microdroplets is known as photonic cross-communication.[2] For example, according to Bragg's law $\lambda = np\cos\theta$, the central cores of the infrared microdroplets exhibit normal reflection wavelength at 880 nm ($\lambda\cos0°$), while the red additional lines exhibit double reflection wavelength at 620 nm ($\lambda\cos45°$) (Figure S12).

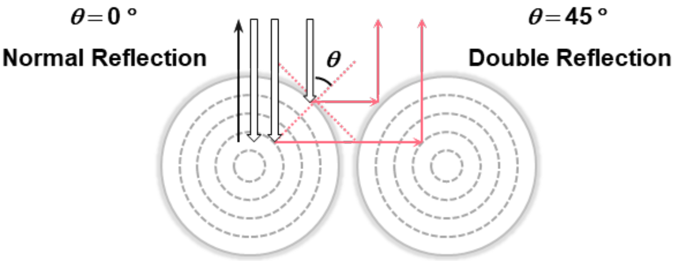

Supplementary Fig. 11 Schematic illustration to show the mechanism of photonic cross-communication caused by double reflection between the neighboring microdroplets.

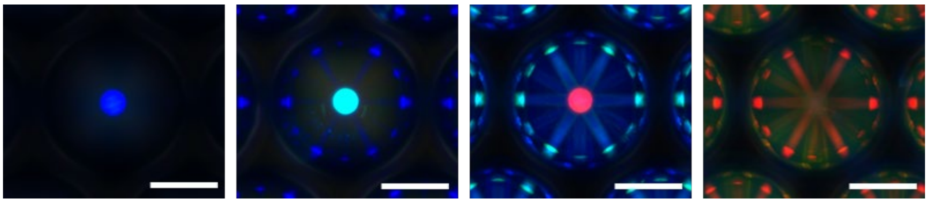

Supplementary Fig. 12 Photonic cross-communication of the central microdroplet in hexagonally close-packed arrays of blue, green, red and infrared monodisperse FCLC microdroplets. The scale bar is 100 μm.

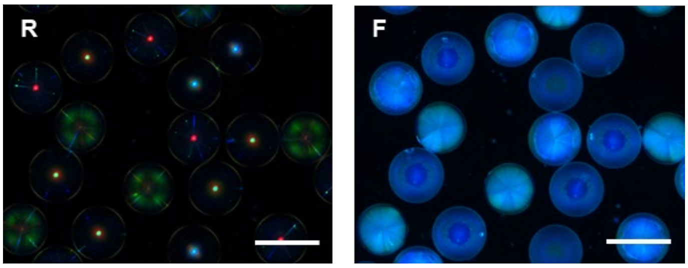

Supplementary Fig. 13 Optical images of the FCLC microdroplets with different pitch lengths taken from a POM in reflection mode (left) and fluorescence mode (right), the diameters of the FCLC microdroplets are ~170 μm. The scale bar is 200 μm.

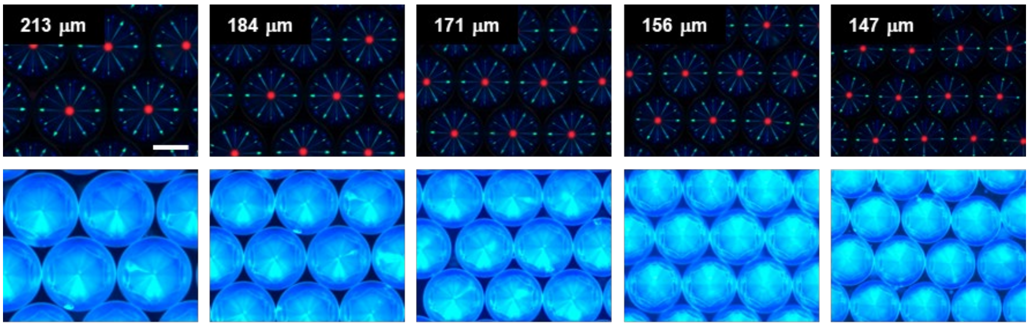

Supplementary Fig. 14 POM images of the red monodisperse FCLC microdroplets with different diameters under white light (top) and upon UV irradiation (right). The scale bar is 100 μm.

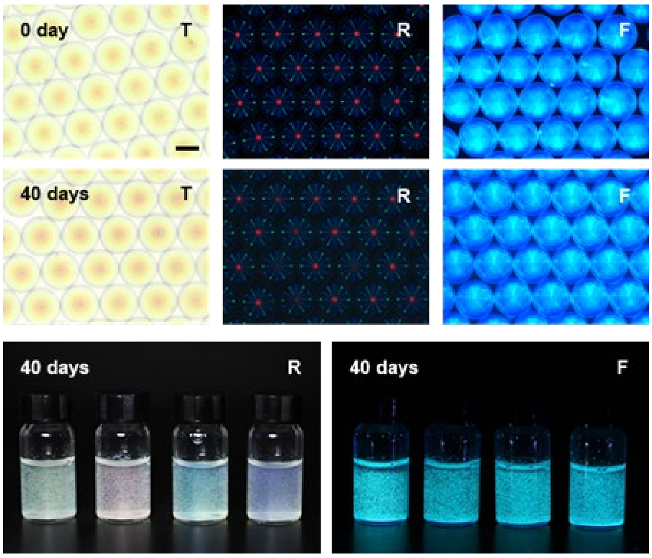

Supplementary Fig. 15 Optical images to show the stability of the FCLC microdroplets taken in transmission mode (left), reflection mode (middle) and fluorescence mode (right) (top). The scale bar is 100 μm. Photographs to show the structural and fluorescent color of the FCLC microdroplets in vials after 40 days (bottom).

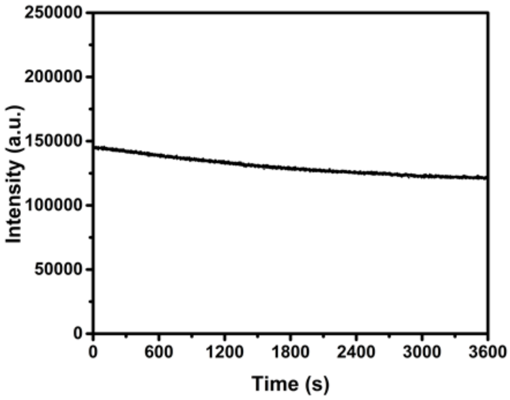

Supplementary Fig. 16 Photobleaching kinetics of monodisperse FCLC microdroplets containing 3 wt% chiral dopants for continuous exposure by 365 nm excitation light. The emission single at 480 nm is collected.

6. The CLC microdroplets without fluorescence

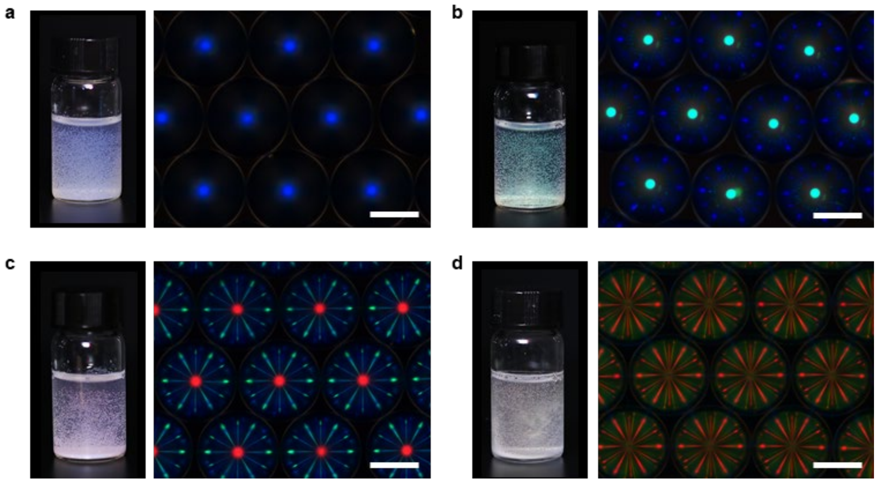

Supplementary Fig. 17 Photographs and POM images of blue (a), green (b), red (c) and infrared (d) monodisperse CLC microdroplets without fluorescence. The scale bar is 100 μm.

7. Preparation of the geminate labels

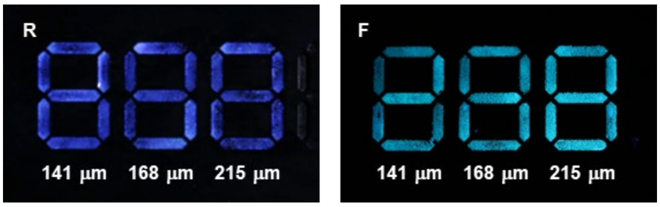

Supplementary Fig. 18 Photographs of a geminate label fabricated by using the blue FCLC microdroplets with different diameters of 141, 168, and 215 μm under white light (left) and upon UV irradiation (right).

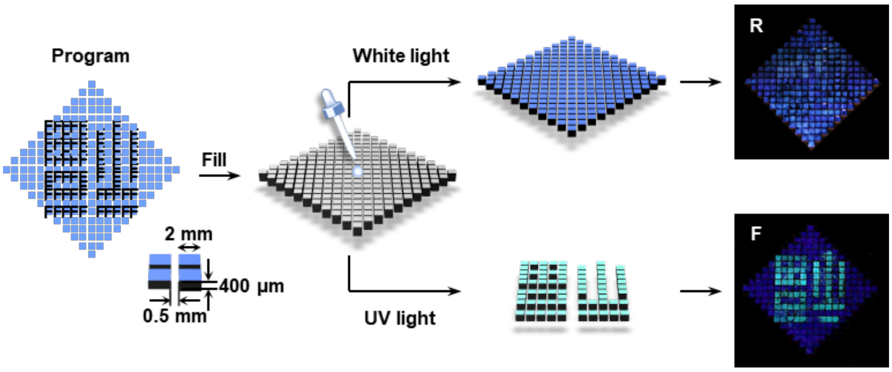

Supplementary Fig. 19 Schematic illustration to show the preparation of a geminate label. The label demonstrates a blue square under white light (R, reflection color) and a cyan inverted Chinese character 'Fu' upon UV irradiation (F, fluorescent color). Each block is 2.0 mm × 2.0 mm × 400 μm, and the distance between two blocks is 0.5 mm.

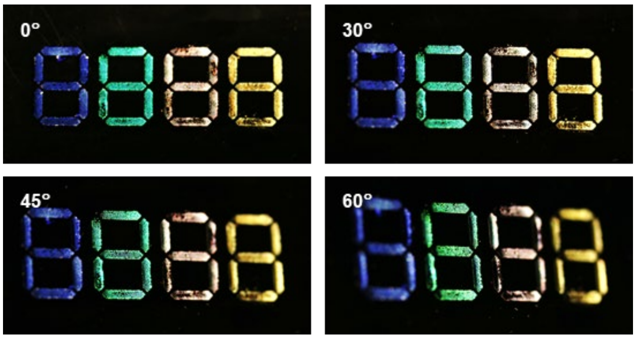

Supplementary Fig. 20 A geminate label composed of the blue, green, red, and infrared FCLC microdroplets observed at different viewing angles (0°, 30°, 45°, and 60°).

8. Supplementary References

[1] Dierking, I. *Textures of Liquid Crystals* (Wiley, Weinheim, Germany 2003).

[2] Noh, J., Liang, H.-L., Drevensek-Olenik, I. & Langerwall, J. P. F. Tuneable multicoloured patterns from photonic cross-communication between cholesteric liquid crystal droplets. *J. Mater. Chem. C* **2**, 806-810 (2014).

9. NMR spectra

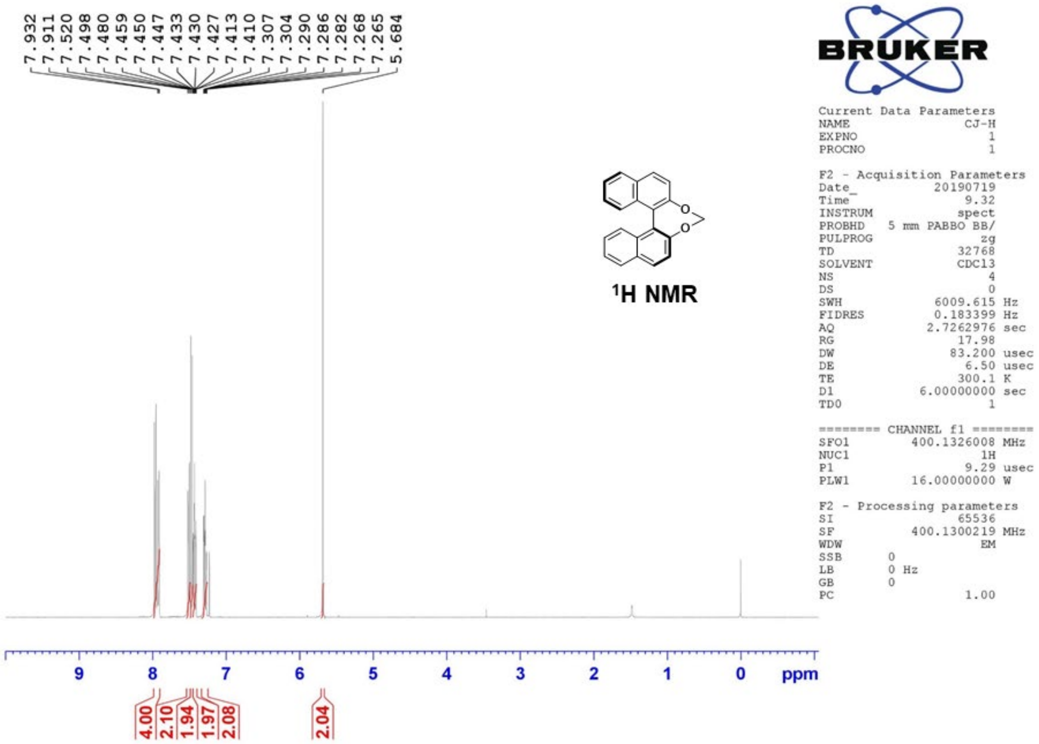

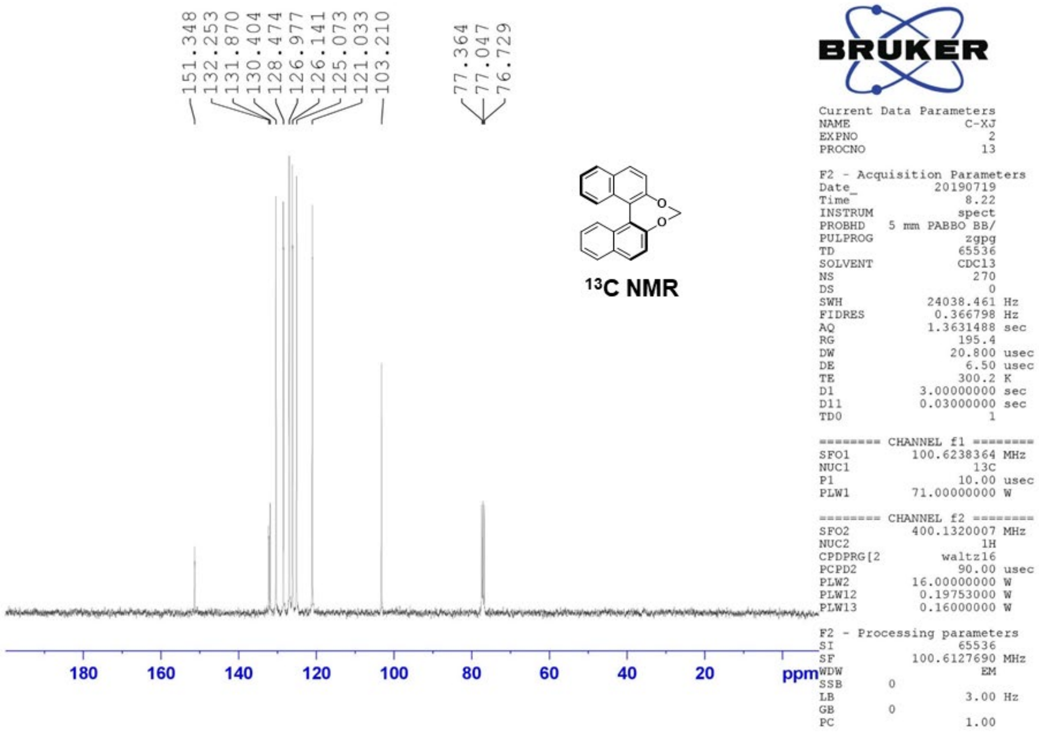

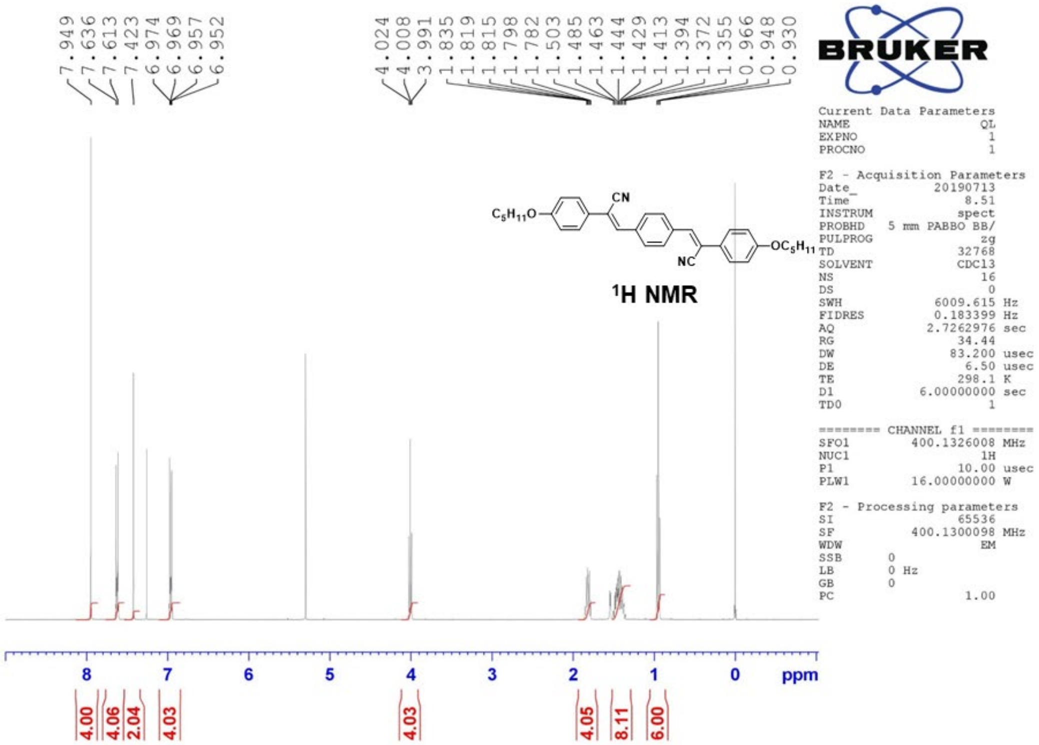
7.949
7.636
7.613
7.423
6.974
6.969
6.957
6.952
4.024
4.008
3.991
1.835
1.819
1.815
1.798
1.782
1.503
1.485
1.463
1.444
1.429
1.413
1.394
1.372
1.355
0.966
0.948
0.930
BRUKER
Current Data Parameters
NAME QL
EXPNO 1
PROCNO 1
F2 - Acquisition Parameters
Date_ 20190713
Time 8.51
INSTRUM spect
PROBHD 5 mm PABBO BB/
PULPROG zg
TD 32768
SOLVENT CDCl3
NS 16
DS 0
SWH 6009.615 Hz
FIDRES 0.183399 Hz
AQ 2.7262976 sec
RG 34.44
DW 83.200 usec
DE 6.50 usec
TE 298.1 K
D1 6.00000000 sec
TD0 1
======== CHANNEL f1 ========
SFO1 400.1326008 MHz
NUC1 1H
P1 10.00 usec
PLW1 16.00000000 W
F2 - Processing parameters
SI 65536
SF 400.1300098 MHz
WDW EM
SSB 0
LB 0 Hz
GB 0
PC 1.00
CN
$C_5H_{11}O$
OC_5H_{11}
NC
^{1}H NMR
8
7
6
5
4
3
2
1
0
ppm
4.00
4.06
2.04
4.03
4.03
4.05
8.11
6.00

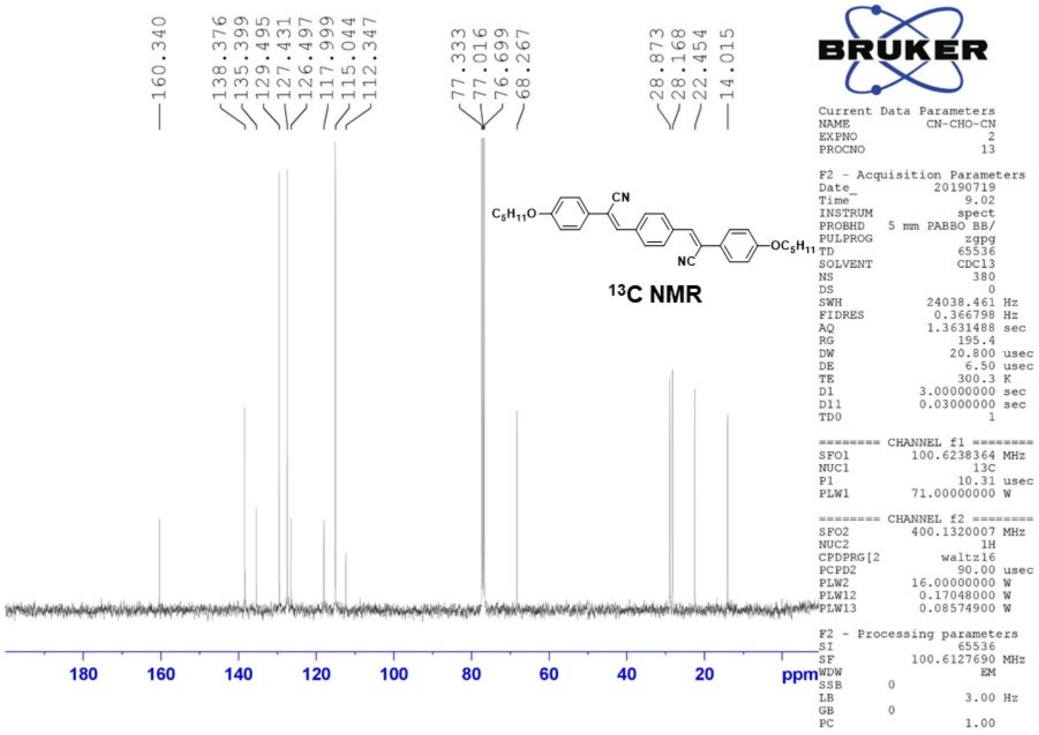
160.340
138.376
135.399
129.495
127.431
126.497
117.999
115.044
112.347
77.333
77.016
76.699
68.267
28.873
28.168
22.454
14.015
BRUKER
Current Data Parameters
NAME CN-CHO-CN
EXPNO 2
PROCNO 13
F2 - Acquisition Parameters
Date_ 20190719
Time 9.02
INSTRUM spect
PROBHD 5 mm PABBO BB/
PULPROG zgpg
TD 65536
SOLVENT CDCl3
NS 380
DS 0
SWH 24038.461 Hz
FIDRES 0.366798 Hz
AQ 1.3631488 sec
RG 195.4
DW 20.800 usec
DE 6.50 usec
TE 300.3 K
D1 3.00000000 sec
D11 0.03000000 sec
TD0 1
======== CHANNEL f1 ========
SFO1 100.6238364 MHz
NUC1 13C
P1 10.31 usec
PLW1 71.00000000 W
======== CHANNEL f2 ========
SFO2 400.1320007 MHz
NUC2 1H
CPDPRG[2 waltz16
PCPD2 90.00 usec
PLW2 16.00000000 W
PLW12 0.17048000 W
PLW13 0.08574900 W
F2 - Processing parameters
SI 65536
SF 100.6127690 MHz
WDW EM
SSB 0
LB 3.00 Hz
GB 0
PC 1.00
CN
$C_5H_{11}O$
OC_5H_{11}
NC
^{13}C NMR
180
160
140
120
100
80
60
40
20
ppm
