# Supplementary material for: Geminate labels programmed by two-tone microdroplets combining structural and fluorescent color
Source: Nat Commun. 2021 Jan 29;12:699. doi: 10.1038/s41467-021-20908-y (PMC7846849; doi:10.1038/s41467-021-20908-y)
Supplement: Supplementary file 2 — Description of Additional Supplementary Files [file 41467_2021_20908_MOESM2_ESM.pdf]

Description of Additional Supplementary Files

File Name: Supplementary Movie 1
Description: The microfluidic fabrication of the FCLC microdroplets under white light and upon UV irradiation. The movie is taken by a digital camera mounted at an optical microscope in transmission mode.

File Name: Supplementary Movie 2
Description: The fluorescent QR code is decrypted by a smartphone upon UV irradiation.

File Name: Supplementary Movie 3
Description: The fluorescent QR code on the computer screen is decrypted by different smartphones.
